# Comparative studies on population genetic structure of two closely related selfing and outcrossing *Zingiber* species in Hainan Island

**DOI:** 10.1038/s41598-019-54526-y

**Published:** 2019-11-29

**Authors:** Rong Huang, Qing-Hua Chu, Guo-Hui Lu, Ying-Qiang Wang

**Affiliations:** 1 Guangdong Provincial Key Laboratory of Biotechnology for Plant Development, School of Life Sciences, South China Normal University, Guangzhou, 510631 China; 2 Guangzhou Key Laboratory of Subtropical Biodiversity and Biomonitoring, School of Life Sciences, South China Normal University, Guangzhou, 510631 China

**Keywords:** Ecology, Evolution

## Abstract

How mating system impacts the genetic diversity of plants has long fascinated and puzzled evolutionary biologists. Numerous studies have shown that self-fertilising plants have less genetic diversity at both the population and species levels than outcrossers. However, the phylogenetic relationships between species and correlated ecological traits have not been accounted for in these previous studies. Here, we conduct a comparative population genetic study of two closely related selfing and outcrossing *Zingiber* species, with sympatric distribution in Hainan Island, and obtain a result contrary to previous studies. The results indicate that selfing *Z*. *corallinum* can maintain high genetic diversity through differentiation intensified by local adaptation in populations across the species’ range. In contrast, outcrossing *Z*. *nudicarpum* preserves high genetic diversity through gene exchange by frequent export of pollen within or among populations. Contrary to expectations, the major portion of genetic variation of outcrossing *Z*. *nudicarpum* may exist among populations, depending on the dispersal ability of pollen and seed. Our results also reveal that the main factor affecting population structure of selfing *Z*. *corallinum* is mountain ranges, followed by a moist climate, while that of outcrossing *Z*. *nudicarpum* is likely moisture, but not mountain ranges, due to gene flow via pollen.

## Introduction

The mating system used by plants is considered to be a major factor affecting the genetic variability of species and how it impacts the genetic diversity of plants has long fascinated and puzzled evolutionary biologists^1–6^. Evolutionary theory predicts that selfing will evolve when two distinct advantages (transmission advantage and reproductive assurance) outweigh the evolutionary costs associated with inbreeding depression and reduced fitness caused by the increased homozygosity of deleterious recessive alleles^7^. Nevertheless, a selfing strategy is expected to come at the cost of reduced genetic variation^8–10^. Numerous studies on genetic variation have shown that within-population diversity is typically reduced in selfing species relative to outcrossing species, but genetic differentiation among populations is strengthened^1,11–14^. When patterns of diversity are examined across a species’ entire range, selfers clearly show lower genetic diversity^1,9,11^, but these previous studies did not take into account the phylogenetic relationship between the species concerned. Comparisons of the genetic structure of species from unrelated taxa may be confounded by the effects of correlated ecological traits or phylogeny^2^. Recent comparative population genetic studies of related plant species have also verified the expectation that genetic variation within populations of selfing plants is lower than that of outcrossing plants, such as *Arabidopsis*^10,15,16^, *Amsinckia*^17^, *Capsella*^6^, *Leavenworthia*^18^, *Lycopersicon*^19^, *Mimulus*^3,20^, *Miscanthus*^21^ and *Shorea*^22^. In most cases, the species level of genetic diversity is also lower in selfing plants, compared to closely related outcrossing species, but less pronounced than within-population diversity^6,21,23^. Contrary to these studies, there are a few reports of the opposite results, namely that species-wide diversity is not significantly different in (predominantly) selfing plants compared with (predominantly) outcrossing relatives^15,24^. Thus, comparisons of closely related species with different mating systems are needed and could contribute to an improved understanding of the effect of mating system on population genetic diversity and structure^3^.

After mating system, environmental and ecological factors (i. e. moisture, soil, light and temperature) are considered the most important factors shaping the spatial genetic structure within species^25–31^. Isolated populations must utilize their inherent evolutionary potential to adapt to the changing environment^32^, resulting in local adaptive divergence. However, it can be difficult to fully understand what drives genetic diversity because its roots lie in a combination of within- and among-population effects and a complex mixture of climatic, historical and geological influences^33^. Comparing patterns of genetic variation in sympatric species that experience the same environmental influences can disentangle the relative influences on a species’ genetic structure due to biological properties of the species versus those linked to habitat features^34^. So far, most comparative studies on the population genetic structure of co-distributed taxa have focused on animals^34^. There are also a few comparative studies of the effects on genetic differentiation of sympatric and allopatric plant distribution^35–40^. However, empirical studies on the impact of mating system on genetic diversity and genetic differentiation of closely related plants with sympatric distribution are rare (e.g. two sympatric *Delphinium* species: selfing *D*. *barbeyi* and outcrossing *D*. *nuttallianum*^2^). In this study, we compare the effect of mating system on genetic diversity and genetic differentiation in two closely related *Zingiber* species with sympatric distribution in Hainan Island, selfing *Z*. *corallinum*^41^ and outcrossing *Z*. *nudicarpum*^42^, using ISSR (inter-simple sequence repeat) data. Both *Zingiber* species are perennial herbs belonging to the sect. *Zingiber* of the genus *Zingiber*^43–46^. We focus on the following questions: (1) whether selfing *Z*. *corallinum* show less genetic diversity than outcrossing *Z*. *nudicarpum*? (2) whether most of the total genetic variation is due to individual differences within populations of outcrossing *Z*. *nudicarpum* as theory predicts? (3) whether there are differences in factors affecting the population genetic structure of *Zingiber* populations that relate to mating system?

## Results

### ISSR polymorphism and genetic diversity

The ISSR polymorphism and genetic diversity data are shown in Table 1. A total of 225 and 338 bands were produced from 10 and 13 selected primers in *Zingiber corallinum* and *Z*. *nudicarpum*, respectively. Of these, 208 (92.44%) and 324 (95.86%) bands were polymorphic, and 13 (5.8%) and 0 were specific, in *Z*. *corallinum* and *Z*. *nudicarpum*, respectively. At species level, Nei’s genetic diversity (*h*) and the Shannon index (*I*) for *Z*. *corallinum* were almost identical to those of *Z*. *nudicarpum* (*h* = 0.2712 vs 0.2723, *I* = 0.4120 vs 0.4194). However, the population values for *h* and *I* in *Z*. *corallimun* were significantly lower than in *Z*. *nudicarpum* (0.0283 vs 0.1135, 0.0440 vs 0.1756). There was marked variation in *h* and *I* among populations of *Z*. *corallimun* (*h* = 0.0110 to 0.0423, CV = 0.37; *I* = 0.0173 to 0.0645, CV = 0.37), but the opposite was true for *Z*. *nudicarpum* (*h* = 0.0937 to 0.1245, CV = 0.10; *I* = 0.1460 to 0.1949, CV = 0.10).

**Table 1:** Comparison of genetic diversity for *Zingiber corallinum* and *Z*. *nudicarpum*.

Species	Population	*PL*	*PPL* (%)	*N*a	*N*e	*h*	*I*	NS
***Z***. ***corallinum***								
	HNWN-D	13	4.59	1.0459	1.0181	0.0110	0.0173	1
	HNQZ	36	12.72	1.1272	1.0545	0.0343	0.0535	1
	HNDZ-L	25	8.83	1.0883	1.0305	0.0196	0.0314	3
	HNDZ-S	29	10.25	1.1025	1.0447	0.0275	0.0426	2
	HNBT	41	14.49	1.1449	1.0565	0.0360	0.0568	2
	HNCJ	27	9.54	1.0954	1.0456	0.0273	0.0417	1
	HNLD	43	15.19	1.1519	1.0699	0.0423	0.0645	2
	Mean	30.6	10.80	1.1080	1.0457	0.0283	0.0440	1.7
	Total	208	92.44	1.9244	1.4526	0.2712	0.4120	13
***Z***. ***nudicarpum***								
	HNWN-X	154	45.56	1.4556	1.2046	0.1241	0.1919	0
	HNLS	139	41.12	1.4112	1.1854	0.1131	0.1753	0
	HNQZ	123	36.39	1.3639	1.1524	0.0937	0.1460	0
	HNDZ-S	162	47.93	1.4793	1.2024	0.1245	0.1949	0
	HNBT	128	37.87	1.3787	1.1855	0.1117	0.1712	0
	HNCJ	134	39.64	1.3964	1.1897	0.1138	0.1743	0
	Mean	140.0	41.42	1.4142	1.1867	0.1135	0.1756	0
	Total	324	95.86	1.9586	1.4494	0.2723	0.4194	0

*PL*: number of polymorphic loci; *PPL*: percentage of polymorphic loci; *N*a: number of observed alleles; *N*e: number of effective alleles; *h*: Nei’s gene diversity; *I*: Shannon’s information index; NS: number of specific bands.

**Figure 1:**
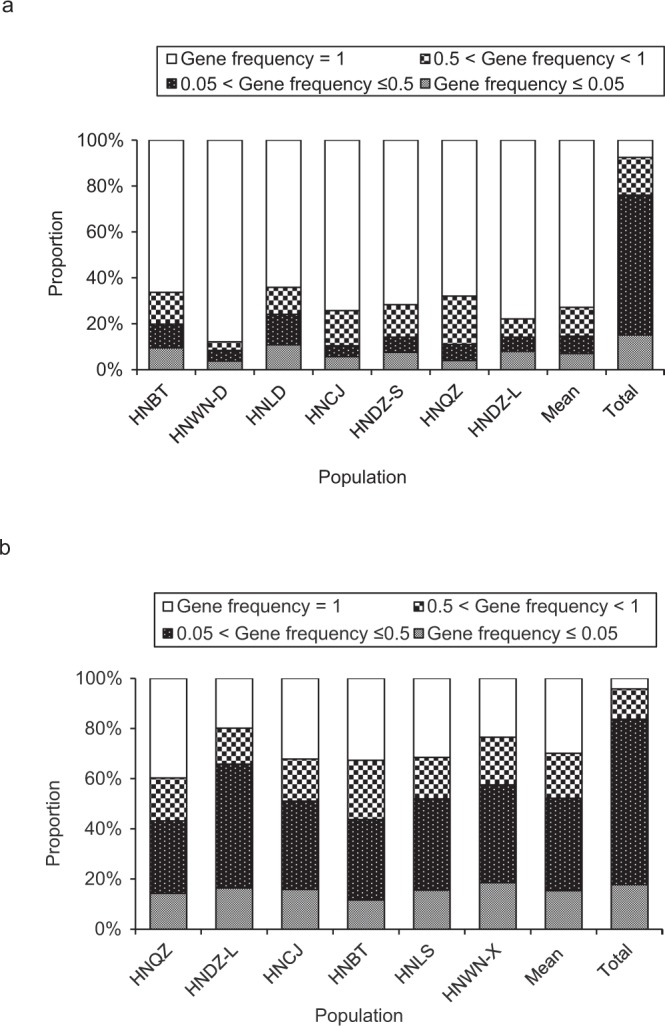
Gene frequency distribution at population level and species level in *Zingiber corallinum* (**a**) and *Z*. *nudicarpum* (**b**).

The patterns of gene frequency distribution are shown in Fig. 1. At species level, low-to-medium gene frequency loci (i.e. found in 5–50% individuals: 5%< gene frequency ≤50%) in *Z*. *corallinum* and *Z*. *nudicarpum* accounted for the highest proportion of amplified fragments, i.e. 60.89% and 65.98%, followed by medium-to-high gene frequency loci (50%< gene frequency <100%) and rare loci (gene frequency ≤5%), i.e. 16.44% and 15.11%, 12.13% and 17.75%, respectively. Common loci (gene frequency = 100%) in *Z*. *corallinum* and *Z*. *nudicarpum* accounted for the lowest proportion (7.56% and 4.14%) of amplified fragments. However, among all populations of *Z*. *corallinum*, common loci accounted for the highest proportion (72.89%) of amplified fragments. Both low-to-medium gene frequency loci (7.56%) and rare loci (7.04%) were less prevalent than medium-to-high gene frequency loci (12.51%). Among all populations of *Z*. *nudicarpum*, low-to-medium gene frequency loci accounted for the highest proportion (36.87%) of amplified fragments. Common loci were more prevalent (29.88%) than medium-to-high gene frequency loci (17.92%) and rare loci (15.32%).

### Genetic differentiation and gene flow

Nei’s genetic differentiation statistics for all populations of the two *Zingiber* species are shown in Supplementary Table S1. The population differentiation value (*G*_ST_) for the populations of *Z*. *corallinum* (0.872) was significantly higher than that of *Z*. *nudicarpum* (0.580), which indicates that 12.8% and 42.0% of total genetic variability were distributed within populations of *Z*. *corallinum* and *Z*. *nudicarpum*, respectively. The level of gene flow (*N*m) among populations of *Z*. *corallinum* and *Z*. *nudicarpum* were estimated to be 0.073 and 0.362, respectively. The result of AMOVA analyses (Table 2) were consistent with the Nei’s genetic differentiation statistics, showing that 91.7% (*Φ*_ST_ = 0.917) and 65.3% (*Φ*_ST_ = 0.653) of the total variation was partitioned among populations of *Z*. *corallinum* and *Z*. *nudicarpum*, respectively. That is, of total genetic variation, only 8.3% was due to individual differences within populations in selfing *Z*. *corallinum*, while 34.7% was attributable to individual differences in outcrossing *Z*. *nudicarpum*.

**Table 2:** Results of analysis of molecular variance (AMOVA) for *Zingiber corallinum* and *Z*. *nudicarpum*.

Species	Source of variation	df	SS	MS	Variance component	Percentage of variation	*Φ*-statistics	*p*
***Z***. ***corallinum***								
	Among populations	6	5824.37	970.73	32.26	91.7%	*Φ*_ST_ = 0.917	0.001
	Within populations	203	592.7	2.92	2.92	8.3%		
***Z***. ***nudicarpum***								
	Among populations	5	5671.43	1134.29	36.86	65.3%	*Φ*_ST_ = 0.653	0.001
	Within populations	176	3446.28	19.58	19.58	34.7%		

df: degrees of freedom; SS: sums of squares; MS: mean of squares; *Φ*_ST_: among-population deviations from Hardy-Weinberg expectations; *p*: the probability of null hypothesis.

### Genetic structure and cluster analysis

**Figure 2:**
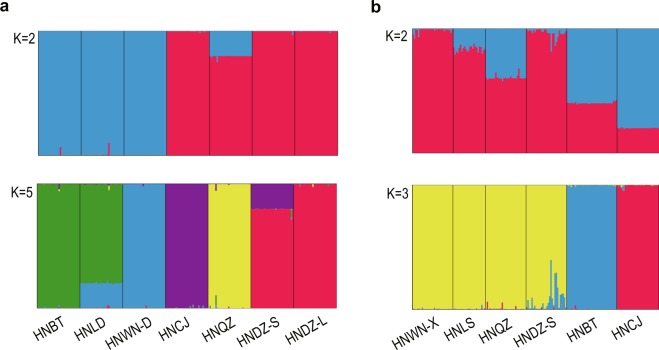
Genetic group structure shown by STRUCTURE analysis for *Zingiber corallinum* (**a**) and *Z*. *nudicarpum*. (**b**) Each vertical bar represents one population and different colors represent different gene pools.

Bayesian genetic analysis performed with STRUCTURE revealed that, with *K* = 2, all populations of *Z*. *corallinum* were assigned to two genetic clusters (Fig. 2a). Except for population HNQZ, all individuals within each population were assigned to the same genetic clusters. At the highest log likelihood (*K* = 5), all populations of *Z*. *corallinum* could be assigned to five genetic clusters (Fig. 2a). Except for two populations (HNLD and HNDZ-S), all individuals within each population were assigned to the same genetic clusters. In HNLD and HNDZ-S, there was a high degree of admixture of two gene pools (HNBT vs HNWN-D, HNCJ vs HNDZ-L, respectively) in all individuals. With *K* = 2, all populations of *Z*. *nudicarpum* were assigned to two genetic clusters (Fig. 2b). Except for population HNWN-X, there was some degree of admixture of two gene pools in all individuals within each population. Given the maximum log likelihood value (*K* = 3), all populations of *Z*. *nudicarpum* were assigned to three genetic clusters (Fig. 2b). All individuals in populations HNWN-X, HNLS, HNQZ and HNDZ-S were assigned to the same genetic cluster, while the populations HNBT and HNCJ each corresponded to a separate genetic cluster. In population HNDZ-S, there was some degree of admixture of the HNBT gene pool in individuals.

**Figure 3:**
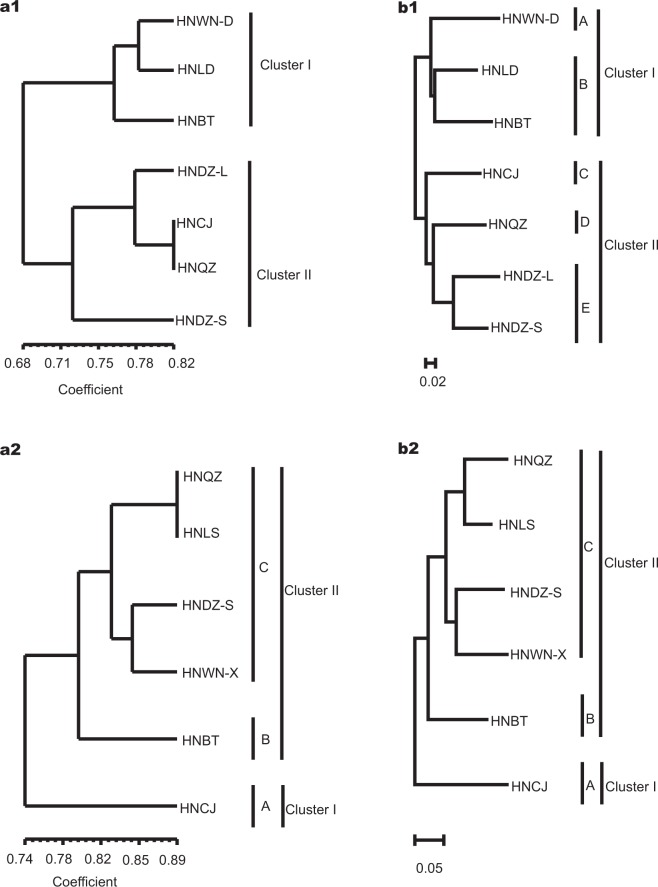
UPGMA dendrogram and unrooted neighbour-joining (NJ) tree of seven populations of *Zingiber corallinum* and six populations of *Z*. *nudicarpum*. (a–UPGMA dendrogram, b–NJ tree, 1–*Z*. *corallinum*, 2–*Z*. *nudicarpum*).

**Figure 4:**
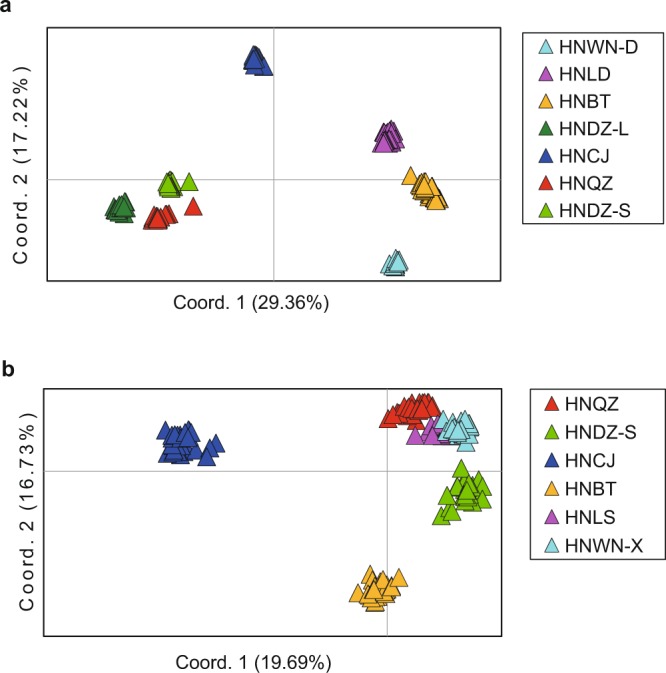
Principal coordinate analysis of 210 individuals from seven populations of Z*ingiber corallinum* (**a**) and 182 individuals from six populations of *Z*. *nudicarpum*. (**b**) Different colors represent different populations.

The UPGMA dendrogram (Fig. 3a1) based on Nei’s genetic identity was broadly consistent with the unrooted neighbor-joining (NJ) tree (Fig. 3b1) based on Nei’s genetic distance in populations of *Z*. *corallinum*. Seven populations of *Z*. *corallinum* were first classified into two clusters (I, II), which comprised three populations (HNWN-D, HNLD, HNBT) and four populations (HNDZ-L, HNDZ-S, HNQZ, HNCJ), respectively. However, the UPGMA dendrogram was in conflict with the NJ tree within the two clusters. In the NJ tree, cluster I further formed two well-resolved clades (A, B), which comprised population HNWN-D and the other two populations (HNBT, HNLD), respectively (Fig. 3b1). Cluster II also formed two further groups with three well-resolved clades (C, D, E), which comprised populations HNCJ, HNQZ and the other two populations (HNDZ-S, HNDZ-L), respectively. Both STRUCTURE analysis (Fig. 2a) and PCoA (Fig. 4a) confirmed the partitioning results of the NJ tree clustering. The UPGMA dendrogram (Fig. 3a2) was consistent with an unrooted neighbor-joining (NJ) tree (Fig. 3b2) in the six populations of *Z*. *nudicarpum*. Six populations were first grouped into two clusters (I, II), with cluster I consisting of clade A only, which comprised population HNCJ. Cluster II further formed two well-resolved clades (B, C), which comprised populations HNBT and the other four populations (HNDZ-S, HNWN-X, HNLS, HNQZ), respectively. STRUCTURE analysis (Fig. 2b) and PCoA (Fig. 4b) revealed a pattern that was consistent with the UPGMA tree and the NJ tree. There was no significant isolation-by-distance relationship across populations of *Z*. *corallinum* (*p* = 0.150) and *Z*. *nudicarpum* (*p* = 0.070) (Supplementary Fig. S1).

## Discussion

Is genetic diversity in a selfing *Zingiber* species lower than in an outcrossing *Zingiber* species? Generally, compared with outcrossing species, selfing species are expected to have lower effective population sizes and recombination rates, leading directly to reduced genetic diversity, increased linkage disequilibrium and increased homozygosity^9^. Numerous studies on genetic diversity among both closely related and unrelated species show that selfing species have less genetic diversity and heterozygosity than outcrossing species at both the population level^13,14,47–49^ and the species level^1,6,9,11,21,23^. In contrast, our results reveal that the level of species-wide genetic diversity of selfing *Z*. *corallinum* was comparable to that of outcrossing *Z*. *nudicarpum* (*h*: 0.2712 vs 0.2723, *I*: 0.4120 vs 0.4194), despite the population genetic diversity in selfing *Z*. *corallinum* being significantly lower than that in outcrossing *Z*. *nudicarpum* (*h*: 0.0283 vs 0.1135, *I*: 0.0440 vs 0.1756). This indicates that selfing *Z*. *corallinum* can maintain a high level of genetic diversity, similar to that of outcrossing species, albeit by using different strategies. The population genetic diversity of selfers is expected to be reduced by a factor of two compared to outcrossers^8,11,49^, but the loss of genetic diversity has been shown to be more severe in a number of comparative studies on closely related selfing and outcrossing species^10,15,49–51^. In the present study, the average population genetic diversity of selfing *Z*. *corallinum* was four times lower than in outcrossing *Z*. *nudicarpum*, consistent with the literature on closely related species. Our results also showed that common loci are very highly represented within populations of *Z*. *corallinum*, indicating that the corresponding individuals are highly homogeneous. The test of multilocus linkage disequilibrium (${\bar{r}}_{{\rm{d}}}$) also showed that mean ${\bar{r}}_{{\rm{d}}}$ of *Z*. *corallinum* was significantly higher than that of *Z*. *nudicarpum* (0.0975 ± 0.0464 vs 0.0314 ± 0.0110, *p* = 0.009). Thus, our study confirms the hypothesis that a selfing species is expected to show reduced genetic diversity within populations and increased homozygosity compared with a related outcrossing species.

Previous studies have shown that outcrossing plants can preserve a degree of genetic diversity through frequent gene flow among populations, while genetic differentiation can effectively be eliminated when gene flow per generation is very low^52,53^. This is reflected in our demonstration that the populations of outcrossing *Z*. *nudicarpum* are highly homogenous, as evidenced by the absence of specific bands in populations across the range of the species. On the contrary, pollen migration among populations is rare in selfing plants and a specific locus that arises in an individual population cannot spread to other populations, resulting in a high level of genetic divergence among populations^47,54^. An increase in genetic differentiation among populations accompanied by a decrease in pollen flow may lead to an increase in species-wide genetic diversity^47,55^. Therefore, selfing plants can still preserve a comparable level of genetic variation across their range, as the various populations may fix different alleles^56^. This is the case for selfing *Z*. *corallinum* in our study. There is a high degree of genetic variation among populations of *Z*. *corallinum* (CV = 0.37) compared with *Z*. *nudicarpum* (CV = 0.10), which indicates that population habitat heterogeneity may result in a higher fluctuation of genetic diversity in selfing *Z*. *corallinum*. Thus, ecological heterogeneity always causes an increase in genetic variation in natural plant populations^57^, and this is preserved in selfing *Z*. *corallinum* due to lack of pollen migration within and among populations. The greater number of specific ISSR bands in selfing *Z*. *corallinum* populations is evidence of this phenomenon, and shows that the populations of *Z*. *corallinum* are highly heterogeneous. Moreover, our result reveals that there is a high differentiation value among populations of selfing *Z*. *corallinum* (*Φ*_ST_ = 0.917) compared with outcrossing *Z*. *nudicarpum* (*Φ*_ST_ = 0.653), which indicates that strong isolation among populations and/or isolation, intensified by local adaptation^3^, leads to fixation of different loci^58^ in populations of selfing *Z*. *corallinum*. The proportion of common loci within populations of selfing *Z*. *corallinum* is significantly higher than in outcrossing *Z*. *nudicarpum*. However, at the species level, the pattern of gene frequency distribution of selfing *Z*. *corallinum* is consistent with that of *Z*. *nudicarpum*, in which common loci account for the lowest proportion and low-to-medium gene frequency loci account for the highest proportion. These results show that there are very different allele frequencies among populations, thus resulting in a high level of species-wide diversity^55^ in selfing *Z*. *corallinum*, like outcossing *Z*. *nudicarpum*. In conclusion, we suggest that a selfing *Zingiber* species can maintain high genetic diversity through isolation intensified by local adaptation in populations across the species’ range, while in contrast an outcrossing *Zingiber* species preserve high genetic diversity by frequent export of pollen within or among populations.

Whether most of the total genetic variation is due to individual differences within populations of an outcrossing *Zingber* species? The pattern of genetic diversity distribution within and among plant populations is determined by two main characteristics, mating system and gene flow^59^. Because pollen migration in selfing plants is rare, there can be a lack of gene flow among populations, thereby increasing genetic differentiation among populations due to genetic drift and fixation of different loci within genetically isolated populations^60,61^. Thus, selfing plants theoretically have lower genetic diversity within populations and higher differentiation among populations^8^, which has been confirmed by many empirical studies^2,4,23,48,49,62,63^. Our result is also consistent with the theoretical prediction that selfing *Z*. *corallinum* harbors only limited genetic variance (8.3%) within populations, but a high degree of differentiation among populations (*Φ*_ST_ = 0.917, *H*_T_ − *H*_S_ = 0.237). On the other hand, outcrossing plants typically should show greater genetic variation within populations and a low level of differentiation among populations^64^. However, our AMOVA analysis revealed that most of the genetic variation in outcrossing *Z*. *nudicarpum* was also found among populations (65.3%), rather than within populations (34.7%). The observed genetic diversity distribution pattern of outcrossing *Z*. *nudicarpum* populations can be attributed to the short distances of pollen movement via parasitic bees^42^ and restricted seed dispersal by gravity (our observation), which is confirmed by the low level of gene flow (*Nm* = 0.362) and relatively high genetic differentiation (*Φ*_ST_ = 0.653, *H*_T_ − *H*_S_ = 0.157) among populations in *Z*. *nudicarpum* compared with that (*Φ*_ST_ = 0.28) in most outcrossing plants^12^. The Mantel tests also show that neither outcrossing *Z*. *nudicarpum* nor selfing *Z*. *corallinum* exhibits a pattern of isolation by distance among populations, suggesting that the stochastic force of genetic drift is much stronger than gene flow in determining the structure of populations^49^ in both the outcrossing *Z*. *nudicarpum* and the selfing *Z*. *corallinum*. We suggest that most of the genetic variation resides among populations in a selfing *Zingiber* species, while the major portion of genetic variation in an outcrossing *Zingiber species* may exist within or among populations, depending on the degree of isolation and the dispersal ability of pollen and seed.

Whether there are differences in factors affecting the population genetic structure of selfing and outcrossing *Zingiber* plants? The results of both NJ and UPGMA analysis reveal a clear pattern of population structure in *Z*. *corallinum*, with two clusters corresponding to two mountain ranges in Hainan, Wuzhi mountain range (cluster I: populations HNWN-D, HNLD and HNBT) and Limu mountain range (cluster II: populations HNDZ-L, HNDZ-S, HNQZ and HNCJ). The two mountain ranges are separated by the Changhua river valley. Our result is consistent with that obtained for two members of the Gesneriaceae (African violet) family endemic to Hainan Island, *Metapetrocosmea peltata*^65^ and *Oreocharis dasyantha*^66^, in which genetic structure is concordant with the isolation pattern of the two mountain ranges, due to very weak gene flow among populations (*Nm* = 0.04). This indicates that topography is the major factor affecting population structure in selfing *Z*. *corallinum* in Hainan island, due to the absent of pollen movement^41^ and restricted seed dispersal by gravity (our observation). In the NJ tree, each cluster (I and II) forms two further groups (clades), which is completely congruent with the climate regionalization delimitation scheme^67^. In cluster I, clade A (population HNWN-D) and clade B (populations HNBT and HNLD) locate in the humid region and the semi-humid region, respectively. In cluster II, one group (clade C, population HNCJ) lies in the semi-humid region, while the other group (including clade D, population HNQZ and clade E, populations HNDZ-L, HNDZ-S) is located in the humid region. This implies that moisture level in the environment also plays an important role in determining patterns of genetic structure in *Z*. *corallinum*. The Bayesian genetic structure and PCoA also confirm the partitioning results of the NJ analysis. We suggest that the major factors affecting population structure in a selfing *Zingiber* species are topography (i.e. mountain range, river valley) and climate (i.e. moisture), which are responsible for the absence of pollen movement (gene flow) and restricted seed dispersal. Both the NJ tree and the UPGMA dendrogram divide six populations of *Z*. *nudicarpum* into three well-resolved clades (A, B, C), whose origin can be attributed to the different climate in the regions they inhabit, i.e. a semi-humid region (clade A, comprising population HNCJ only), a semi-humid and humid boundary region (clade B, comprising population HNBT only) and a humid region (clade C, comprising populations HNDZ-S, HNQZ, HNLS and HNWN-X) in Hainan island. Bayesian genetic structure analysis and PCoA also confirm the partitioning results of the UPGMA clustering and the NJ tree. Together, these data demonstrate that population structure in outcrossing *Z*. *nudicarpum* is likely to be mainly driven by climate (i.e. moisture) and topography (i.e. mountain range, river valley) seems not to be the influence factor of genetic structure like selfing *Z*. *corallinum*, due to gene flow via pollen among neighbouring populations in outcrossing *Z*. *nudicarpum*.

## Methods

### Study species and population sampling

*Zingiber corallinum* and *Z*. *nudicarpum* are two closely related^43–46^ perennial herbs with contrasting mating systems. *Z*. *corallinum* is predominantly self-pollinated^41^ and *Z*. *nudicarpum* is usually cross-pollinated by a parasitic bee^42^. Seeds of both species spread mainly by gravity (our observation). Z. *corallinum* is endemic in south China and *Z*. *nudicarpum* is mainly distributed in south China, Vietnam and Thailand. Both species show sympatric distribution in Hainan Island, China.

**Figure 5:**
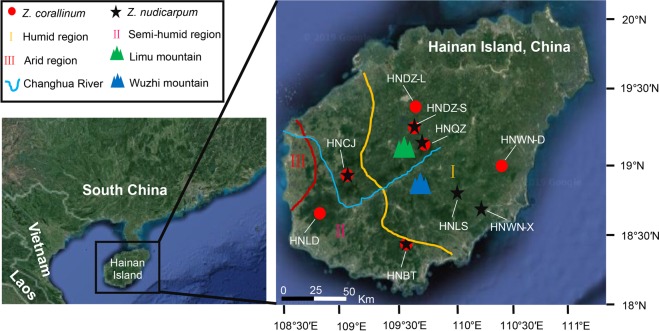
Geographic distribution of sampled populations of *Zingiber corallinum* and *Z*. *nudicarpum*. The area to the right of the yellow line is the humid region (I), the area between the yellow line and the red line is the semi-humid region (II), and the area to the left of the red line is the arid region (III). The climate regionalization scheme of Hainan Island is according to Che *et al*.^67^. The original satellite imagery was obtained from Google Map (Map data ©2019 Google; https://maps.google.com/), and modified with Adobe Illustrator CS6 (Adobe Systems Incorporated, San Jose, CA, USA).

To investigate genetic variation and population structure, 210 individuals from seven populations of *Z*. *corallinum* and 182 individuals from six populations of *Z*. *nudicarpum* were collected from Hainan Island (Fig. 5 and Table 3). The 13 populations of *Z*. *corallinum* and *Z*. *nudicarpum* are distributed in different mountain ranges and regions that differ in humidity. Four populations of *Z*. *corallinum* (HNCJ, HNQZ, HNDZ-S, HNDZ-L) and three populations of *Z*. *nudicarpum* (HNCJ, HNQZ, HNDZ-S) are located in the Limu mountain range, while three populations of *Z*. *corallinum* (HNLD, HNBT, HNWN-D) and three populations of *Z*. *nudicarpum* (HNBT, HNLS, HNWN-X) are located in the Wuzhi mountain range. Four populations of *Z*. *corallinum* (HNQZ, HNDZ-S, HNDZ-L, HNWN-D) and four populations of *Z*. *nudicarpum* (HNQZ, HNDZ-S, HNLS, HNWN-X) lie in a humid region, and the five remaining populations (*Z*. *corallinum*: HNCJ, HNBT, HNLD; *Z*. *nudicarpum*: HNCJ, HNBT) are located in a semi-humid region. Except for population HNLS of *Z*. *nudicarpum*, in which fewer individuals were available (Table 3), at least 30 individuals within each population were sampled. Spatial distances between neighbouring samples were at least 5 m to increase the possibility of detecting genetic variation within each population. Leaves were collected in the field and stored in silica gel.

**Table 3:** Details of sampled populations of *Zingiber corallinum* (ZC) and *Z*. *nudicarpum* (ZN).

Populations	Species	Location	Latitude (°N)	Longitude (°E)	Altitude (m)	Sample size	Mountain ranges	Climate region*
HNWN-D	ZC	Dongling, Wanning, Hainan	19°00′51″	110°24′53″	40	30	Wuzhi	Humid region
HNWN-X	ZN	Xinglong, Wanning, Hainan	18°47′39″	110°08′41″	108	30	Wuzhi	Humid region
HNLS	ZN	Diaoluoshan, Lingshui, Hainan	18°50′51″	109°59′20″	602	24	Wuzhi	Humid region
HNBT	ZC/ZN	Luokui, Baoting, Hainan	18°25′01″	109°34′09″	136	30/37	Wuzhi	Semi-humid region
HNLD	ZC	Jianfengling, Ledong, Hainan	18°42′04″	108°48′53″	138	30	Wuzhi	Semi-humid region
HNQZ	ZC/ZN	Limushan, Qiongzhong, Hainan	19°10′16″	109°44′55″	702	30/30	Limu	Humid region
HNDZ-L	ZC	Lianhuashan, Danzhou, Hainan	19°26′35″	109°39′44″	225	30	Limu	Humid region
HNDZ-S	ZC/ZN	Sanya, Danzhou, Hainan	19°15′52″	109°39′36″	298	30/30	Limu	Humid region
HNCJ	ZC/ZN	Bawangling, Changjiang, Hainan	18°47′39″	109°09′04″	613	30/31	Limu	Semi-humid region
Total	ZC/ZN					210/182		

*The climate regionalization scheme of Hainan Island is according to Che *et al*.^67^.

### DNA extraction and ISSR-polymerase chain reaction (PCR)

DNA was extracted from leaf tissues following the modified CTAB method described by Doyle and Doyle^68^, and was dissolved in double-distilled water. DNA concentration and quality were checked with a Nano-100 spectrophotometer and by 0.8% agarose gel electrophoresis. ISSR-PCR was performed in a Bio-Rad T100 Thermal Cycler with the following profile: initial denaturation at 95 °C for 5 min, 39 cycles of denaturation at 94 °C for 45 s, annealing for 45 s, extension at 72 °C for 90 s, with a final extension at 72 °C for 10 min. Ten and thirteen selected primers were used with the DNA samples of *Z*. *corallinum* and *Z*. *nudicarpum*, respectively (Supplementary Table S2). PCR was carried out in a total volume of 20 μL, containing 40 ng template DNA, 2.5/2.0 μL 10 × buffer, 1.50/1.00 mmol Mg^2+^, 0.15/0.20 mmol dNTPs, 0.4/0.6 μmol primer, 2.0 U of Taq polymerase and double-distilled water. Negative control reactions without template DNA were also included to verify the absence of contamination. PCR products were separated in 1.8% agarose gels stained in 0.5× TBE buffer with a 100 bp ladder and photographed using a gel documentation system (Bio-Rad GelDoc XR^+^).

The images of the gels were analysed using Image Lab Software (Bio-Rad) to score for the presence (1) or absence (0) of bands and to assign a fragment size to each band. The presence or absence of bands was further confirmed by eye. To ensure the results were reproducible, duplicate PCR amplifications were performed and only clear and reproducible bands were scored.

### Data analysis

The presence/absence data matrix was analyzed in POPGENE v1.31^69^ to estimate percentage of polymorphic loci (*PPL*), number of observed alleles (*N*a), number of effective alleles (*N*e), Nei’s gene diversity (*h*), Shannon’s information index (*I*), and coefficient of genetic differentiation (*G*_ST_). To explore the partitioning of genetic variation and *Φ* value, analysis of molecular variance (AMOVA) was performed in GenAlEx v6.502^70^ based on 999 permutations. Gene flow among populations was estimated indirectly based on the formula: Nm = 0.5(1-*G*_ST_)/*G*_ST_^71^. Bayesian cluster analysis was implemented in the program STRUCTURE 2.1^72^ and was used to assign an individual to *K* genetic clusters with five runs each comprising a burn-in length of 100,000 and a run length of 1,000,000 Markov chain Monte Carlo (MCMC) replications under the admixture model. The optimal value of K was calculated according to the method of Evanno *et al*.^73^. To reveal the genetic relationship between populations, Nei’s genetic identity matrix was used as input for a cluster analysis by the unweighted pair-group method of averages (UPGMA) to generate a dendrogram in NTSYSpc-2.10^74^. The program MEGA v7^75^ was implemented to generate a dendrogram from Nei’s genetic distances with the neighbour-joining algorithm. Principal coordinate analysis (PCoA) was performed as an alternative means of detecting and visualizing the genetic structure implemented in GenAlEx. Mantel tests implemented in GenAlEx were performed to analyze the effects of geographical distance on genetic structure. Moreover, multilocus linkage disequilibrium was also estimated using the index of ${\bar{r}}_{{\rm{d}}}$ ^76^. Calculation of statistics and tests of significance by randomization (1000) were implemented in the program Multilocus v1.2 (http://www.bio.ic.uk/evolve/software/multilocus).

## Supplementary information

Supplementary Information

## References

[1] Hamrick JL, Godt MJW (1996). Effects of life history traits on genetic diversity in plant species. Philos. Trans. R. Soc. Lond. B Biol. Sci..

[2] Williams CF, Ruvinsky J, Scott PE, Hews DK (2001). Pollination, breeding system, and genetic structure in two sympatric *Delphinium* (Ranunculaceae) species. Am. J. Bot..

[3] Charlesworth D (2003). Effects of inbreeding on the genetic diversity of populations. Philos. Trans. R. Soc. Lond. B Biol. Sci..

[4] Song BH, Clauss MJ, Pepper A, Mitchell-Olds T (2005). Geographic patterns of microsatellite variation in *Boechera stricta*, a close relative of *Arabidopsis*. Mol. Ecol..

[5] Duminil J (2007). Can population genetic structure be predicted from life-history traits?. Am. Nat..

[6] St Onge KR, Källman T, Slotte T, Lascoux M, Palmé AE (2011). Contrasting demographic history and population structure in *Capsella rubella* and *Capsella grandiflora*, two closely related species with different mating systems. Mol. Ecol..

[7] Foxe JP (2009). Recent speciation associated with the evolution of selfing in. Capsella. Proc. Natl. Acad. Sci. USA.

[8] Nordborg M (2000). Linkage disequilibrium, gene trees and selfing: an ancestral recombination graph with partial self-fertilization. Genetics.

[9] Glémin S, Bazin E, Charlesworth D (2006). Impact of mating systems on patterns of sequence polymorphism in flowering plants. Proc. Biol. Sci..

[10] Foxe JP, Stift M, Tedder A, Haudry A, Mable WBK (2010). Reconstructing origins of loss of self-incompatibility and selfing in north American A*rabidopsis lyrata*: a population genetic context. Evolution.

[11] Hamrick, J. L. & Godt, M. J. W. Allozyme diversity in plant species in *Plant population genetics*, *breeding and genetic resources* (eds Brown, A. H. D., Clegg, M. T., Kahler, A. L. & Weir, B. S.) 43-63 (Sinauer Associates, 1989).

[12] Nybom H, Bartish IV (2000). Effects of life history traits and sampling strategies on genetic diversity estimates obtained with RAPD markers in plants. Perspect. Plant Ecol..

[13] Nybom H (2004). Comparison of different nuclear DNA markers for estimating intraspecific genetic diversity in plants. Mol. Ecol..

[14] Reisch C, Bernhardt-Römermann M (2014). The impact of study design and life history traits on genetic variation of plants determined with AFLPs. Plant Ecol..

[15] Savolainen O, Langley CH, Lazzaro BP, Fréville H (2000). Contrasting patterns of nucleotide polymorphism at the alcohol dehydrogenase locus in the outcrossing *Arabidopsis lyrata* and the selfing *Arabidopsis thaliana*. Mol. Biol. Evol..

[16] Mable BK, Adam A (2007). Patterns of genetic diversity in outcrossing and selfing populations of Arabidopsis lyrata. Mol. Ecol..

[17] Pérusse JR, Schoen DJ (2004). Molecular evolution of the GapC gene family in *Amsinckia spectabilis* populations that differ in outcrossing rate. J. Mol. Evol..

[18] Liu F, Zhang L, Charlesworth D (1998). Genetic diversity in Leavenworthia populations with different inbreeding levels. Proc. R. Soc. Lond. B. Biol. Sci..

[19] Baudry E, Kerdelhue C, Innan H, Stephan W (2001). Species and recombination effects on DNA variability in the tomato genus. Genetics.

[20] Fenster CB, Ritland K (1992). Chloroplast DNA and isozyme diversity in two Mimulus species (Scrophulariaceae) with contrasting mating systems. Am. J. Bot..

[21] Chiang YH, Schaal BA, Chou CH, Huang S, Chiang TY (2003). Contrasting selection modes at the Adh1 locus in outcrossing *Miscanthus sinensi*s vs. inbreeding *Miscanthus condensatus* (Poaceae). Am. J. Bot..

[22] Kevin KS, Lee SL, Koh CL (2004). Spatial structure and genetic diversity of two tropical tree species with contrasting breeding systems ad different ploidy levels. Mol. Ecol..

[23] Sweigart AL, Willis JH (2003). Patterns of nucleotide diversity in two species of *Mimulus* are affected by mating system and asymmetric introgression. Evolution.

[24] Awadalla P, Ritland K (1997). Microsatellite Variation and Evolution in the *Mimulus guttatus* Species complex with Contrasting Mating Systems. Mol. Biol. Evol..

[25] Kozak KH, Graham CH, Wiens JJ (2008). Integrating GIS-based environmental data into evolutionary biology. Trends Ecol. Evol..

[26] Alvarez N (2009). History or ecology? Substrate type as a major driver of spatial genetic structure in Alpine plants. Ecol. Lett..

[27] Temunović M (2012). Environmental heterogeneity explains the genetic structure of continental and mediterranean populations of *Fraxinus angustifolia* Vahl. Plos One.

[28] Zellmer AJ, Hanes MM, Hird SM, Carstens BC (2012). Deep phylogeographic structure and environmental differentiation in the carnivorous plant *Sarracenia alata*. Syst. Biol..

[29] Zink RM (2014). Homage to Hutchinson, and the role of ecology in lineage divergence and speciation. J. Biogeogr..

[30] Paz A, Ibáñez R, Lips KR, Crawford AJ (2015). Testing the role of ecology and life history in structuring genetic variation across a landscape: A trait-based phylogeographic approach. Mol. Ecol..

[31] Xu B (2017). Population genetic structure is shaped by historical, geographic, and environmental factors in the leguminous shrub *Caragana microphylla* on the Inner Mongolia Plateau of China. BMC Plant Biol..

[32] Jonsson M, Bertilsson M, Ehrlén J, Lönn M (2008). Genetic divergence of climatically marginal populations of *Vicia pisiformis* on the Scandinavian Peninsula. Hereditas.

[33] Hague MTJ, Routman EJ (2016). Does population size affect genetic diversity? A test with sympatric lizard species. Heredity.

[34] Lecompte E, Bouanani MA, Thoisy BD, Crouau-Roy B (2017). How do rivers, geographic distance, and dispersal behavior influence genetic structure in two sympatric new world monkeys?. Am. J. Primatol..

[35] Palme AE, Su Q, Palsson S, Lascoux M (2004). Extensive sharing of chloroplast haplotypes among European birches indicates hybridization among *Betula pendula*, *B*. *pubescens* and *B*. *nana*. Mol. Ecol..

[36] Sullivan JP, Lavoué S, Arnegard ME, Hopkins CD (2004). AFLPs resolve phylogeny and reveal mitochondrial introgression within a species flock of African electric fish (Mormyroidea: Teleostei). Evolution.

[37] Behm JE, Ives AR, Boughman JW (2010). Breakdown in postmating isolation and the collapse of a species pair through hybridization. Am. Nat..

[38] McKinnon G, Smith J, Potts B (2010). Recurrent nuclear DNA introgression accompanies chloroplast DNA exchange between two eucalypt species. Mol. Ecol..

[39] Mehner T (2010). Genetic population structure of sympatric and allopatric populations of Baltic ciscoes (*Coregonus albula* complex, Teleostei, Coregonidae). BMC Evol. Biol..

[40] Wang J, Abbott RJ, Ingvarsson PK, Liu JQ (2014). Increased genetic divergence between two closely related fir species in areas of range overlap. Ecol. Evol..

[41] Wu, W. H. Studies on breeding systems of two species in *Zingiber* (Zingiberaceae). Master’s Thesis, South China Normal University, Guangzhou (2008).

[42] Tan, B. Studies on pollination biology in *Zingiber* (Zingiberaceae). Master’s Thesis, South China Normal University, Guangzhou (2010).

[43] Wu, T. L. & Chen, S. Z. *Flora of China*. Vol.16. (Science Press, 1981).

[44] Fang D (1982). Some new taxa of Zingiberaceae from Guangxi. (In Chinese with English abstract). Guihaia.

[45] Wu, T. L. & Larsen, K. *Flora of China*. Vol. 24. (Science Press and Missouri Botanical Garden Press, 2000).

[46] Theerakulpisut P (2012). Phylogeny of the genus *Zingiber* (Zingiberaceae) based on nuclear ITS sequence data. Kew Bulletin.

[47] Ingvarsson PK (2002). A metapopulation perspective on genetic diversity and differentiation in partially self-fertilizing plants. Evolution.

[48] Eschmann-Grupe G, Neuffer B, Hurka H (2004). Extent and structure of genetic variation in two colonizing *Diplotaxis* species (Brassicaceae) with contrasting breeding systems. Plant Syst. Evol..

[49] Pettengill JB, Briscoe Runquist RD, Moeller DA (2016). Mating system divergence affects the distribution of sequence diversity within and among populations of recently diverged subspecies of *Clarkia xantiana* (Onagraceae). Am. J. Bot..

[50] Charlesworth, D. & Pannell, J. R. Mating Systems and population genetic structure in the light of coalescent theory in *Integrating ecology and evolution in a spatial context* (eds Silvertown, J. & Antonovics, J.) 73–96 (Blackwell Science, 2001).

[51] Ness RW, Wright SI, Barrett SCH (2010). Mating-system variation, demographic history and patterns of nucleotide diversity in the tristylous plant *Eichhornia paniculata*. Genetics.

[52] Jacquemyn H, Vandepitte K, Brys R, Honnay O, Roldán-Ruiz I (2007). Fitness variation and genetic diversity in small, remnant populations of the food deceptive orchid *Orchis purpurea*. Biol. Conserv..

[53] Ohhayashi K, Hodoki Y, Kondo NI, Kunii H, Shimada M (2017). A massive tsunami promoted gene flow and increased genetic diversity in a near threatened plant species. Sci. Rep..

[54] Pannell JR, Charlesworth B (2000). Effects of metapopulation processes on measures of genetic diversity. Philos. T. R. Soc. B.

[55] Charlesworth B, Nordborg M, Charlesworth D (1997). The effects of local selection, balancing polymorphism and background selection on equilibrium patterns of genetic diversity in subdivided populations. Genet. Res..

[56] Frankham, R., Briscoe, D. A. & Ballou, J. D. *Introduction to conservation genetics* (Cambridge University Press 2002).

[57] Nevo ER, Noy R, Lavie B, Beiles A, Muchtar S (1986). Genetic diversity and resistance to pollution. Biol. J. Linn. Soc..

[58] Owuor ED, Fahima T, Beharav A, Korol A, Nevo E (1999). RAPD divergence caused by microsite edaphic selection in wild barley. Genetica.

[59] Hahn CZ, Michalski SG, Durka W (2017). Gene flow in, and mating system of, *Rhododendron simsii* in a nature reserve in subtropical China. Nord. J. Bot..

[60] Culley TM, Wolfe AD (2001). Population genetic structure of the cleistogamous plant species *Viola pubescens* Aiton (Violaceae), as indicated by allozyme and ISSR molecular markers. Heredity.

[61] Duminil J, Hardy OJ, Petit RJ (2009). Plant traits correlated with generation time directly affect inbreeding depression and mating system and indirectly genetic structure. BMC Evol. Biol..

[62] Zhang L, Li QJ, Li HT, Chen J, Li DZ (2006). Genetic diversity and geographic differentiation in *Tacca chantrieri* (Taccaceae): an autonomous selfing plant with showy floral display. Ann. Bot..

[63] Koelling VA, Hamrick JL, Mauricio R (2011). Genetic diversity and structure in two species of *Leavenworthia* with self-incompatible and self-compatible populations. Heredity.

[64] Honnay O, Jacquemyn H (2007). Susceptibility of common and rare plant species to the genetic consequences of habitat fragmentation. Conserv. Biol..

[65] Li, G., Ling, S. J., Chen, W. F., Ren, M. X. & Tang, L. Effects of geographic isolation caused by Changhua River on genetic diversity of Hainan-endemic *Metapetrocosmea peltata* (Gesneriaceae). (In Chinese with English abstract) *Guihaia*, 10.11931/guihaia.gxzw201808024 (2019).

[66] Xing EN, Xu ST, Ren MX (2018). Age Structure and Gene flows of Fine-scale Populations of *Oreocharis dasyantha* (Gesneriaceae), in Alpine Herb Endemic to Hainan Island. (In Chinese with English abstract). J. Trop. Biol..

[67] Che XF, Zhang JH, Huang HJ, Liu SJ, Zhang MJ (2014). Climate Regionalization in Hainan Island. (In Chinese with English abstract). Chin. J. Trop. Agric..

[68] Doyle JJ, Doyle JL (1987). A rapid DNA isolation procedure for small quantities of fresh leaf material. Phytochem. Bull..

[69] Yeh, F. C. *et al*. POPGENE, *the user friendly shareware for population genetic analysis*. Molecular Biology and Biotechnology Center. University of Alberta, Edmonton (1997).

[70] Peakall R, Smouse PE (2012). GenAlEx 6.5: genetic analysis in Excel. Population genetic software for teaching and research-an update. Bioinformatics.

[71] McDermott JM, McDonald BA (1993). Gene flow in plant pathosystems. Ann. Rev. Phytopathol..

[72] Pritchard JK, Stephens M, Donnelly P (2000). Inference of population structure using multilocus genotype data. Genetics.

[73] Evanno G, Regnaut S, Goudet J (2005). Detecting the number of clusters of individuals using the software STRUCTURE: a simulation study. Mol. Ecol..

[74] Rohlf, F. J. NTSYS-pc, version 2.10. Exeter Software, Setauket, New York 2000.

[75] Kumar S, Stecher G, Tamura K (2016). MEGA7: molecular evolutionary genetics analysis version 7.0 for bigger datasets. Mol. Biol. Evol..

[76] Agapow PM, Burt A (2001). Indices of multilocus linkage disequilibrium. Mol. Ecol. Notes..

