# Supplementary material for: Comparative studies on population genetic structure of two closely related selfing and outcrossing Zingiber species in Hainan Island
Source: Sci Rep. 2019 Nov 29;9:17997. doi: 10.1038/s41598-019-54526-y (PMC6884562; doi:10.1038/s41598-019-54526-y)

Comparative studies on population genetic structure of two closely related selfing and outcrossing *Zingiber* species in Hainan Island

Rong Huang[1], Qing-Hua Chu [1], Guo-Hui Lu[1, 2] & Ying-Qiang Wang[1, 2*]

1 Guangdong Provincial Key Laboratory of Biotechnology for Plant Development, School of Life Sciences, South China Normal University, Guangzhou 510631, China

2 Guangzhou Key Laboratory of Subtropical Biodiversity and Biomonitoring, School of Life Sciences, South China Normal University, Guangzhou 510631, China

* Corresponding author: Ying-Qiang Wang (wangyq@scnu.edu.cn)

SUPPLEMENTARY TABLES AND FIGURE

Supplementary Table S1. Genetic differentiation statistics among populations of *Zingiber corallinum* and *Z. nudicarpum*.

	H_T	H_S	G_{ST}	Nm
Z. corallinum				
	0.2712	0.0346	0.872	0.073
Z. nudicarpum				
	0.2704	0.1135	0.580	0.362

H_T: total population diversity; H_S: average within-population diversity; G_{ST}: population differentiation; Nm: gene flow.

Supplementary Table S2. Attributes of ISSR primers of *Zingiber corallinum* (ZC) and *Z. nudicarpum* (ZN) used in the present study.

Primer	Sequence 5 ́to 3 ́	*T*m (°C)	SR (bp)	NT	NP	Species
807	$(AG)_8T$	47	350-1500	17	16	ZN
808	$(AG)_8C$	58	150-2000	25	24	ZN
810	$(GA)_8T$	49/53	200-1700/200-1600	24/26	22/26	ZC/ZN
811	$(GA)_8T$	50/52	200-1800/150-1900	21/26	20/24	ZC/ZN
834	$(AG)_8Y^*T$	53/50	240-2150/250-1900	28/23	26/23	ZC/ZN
835	(AG)8Y*C	58	170-2000	30	29	ZN
841	$(GA)_8Y^*C$	55/52	100-1900/200-1900	18/23	15/22	ZC/ZN
847	$(CA)_8R^*C$	52/55	240-2100/250-1800	17/25	16/25	ZC/ZN
857	$(AC)_8Y^*G$	50/58	250-2000/210-2300	20/35	19/35	ZC/ZN
884	$HBH^*(AG)_7$	48/56	180-1900/220-2200	33/28	33/26	ZC/ZN
887	$DVD^*(TC)_7$	50/56	320-2100/390-2100	22/24	19/20	ZC/ZN
888	$BDB^*(CA)_7$	55/56	200-1700/240-1900	20/30	17/29	ZC/ZN
889	$DBD^*(AC)_7$	49/55	250-1900/180-1900	22/26	21/25	ZC/ZN
Total	-	-	100-2150/150-2300	225/338	208/324	ZC/ZN

*B = (C, G, T), D = (A, G, T), R = (A, T), V = (A, C, G), Y = (C, G), H = (A, C, T).

*T*m: annealing temperature; SR: size range of amplified fragments; NT: number of total bands; NP: number of polymorphic bands.

Supplementary Figure S1. Correlation between geographical distance and Nei's genetic distance: (a) *Zingiber corallinum*; (b) *Z. nudicarpum*.

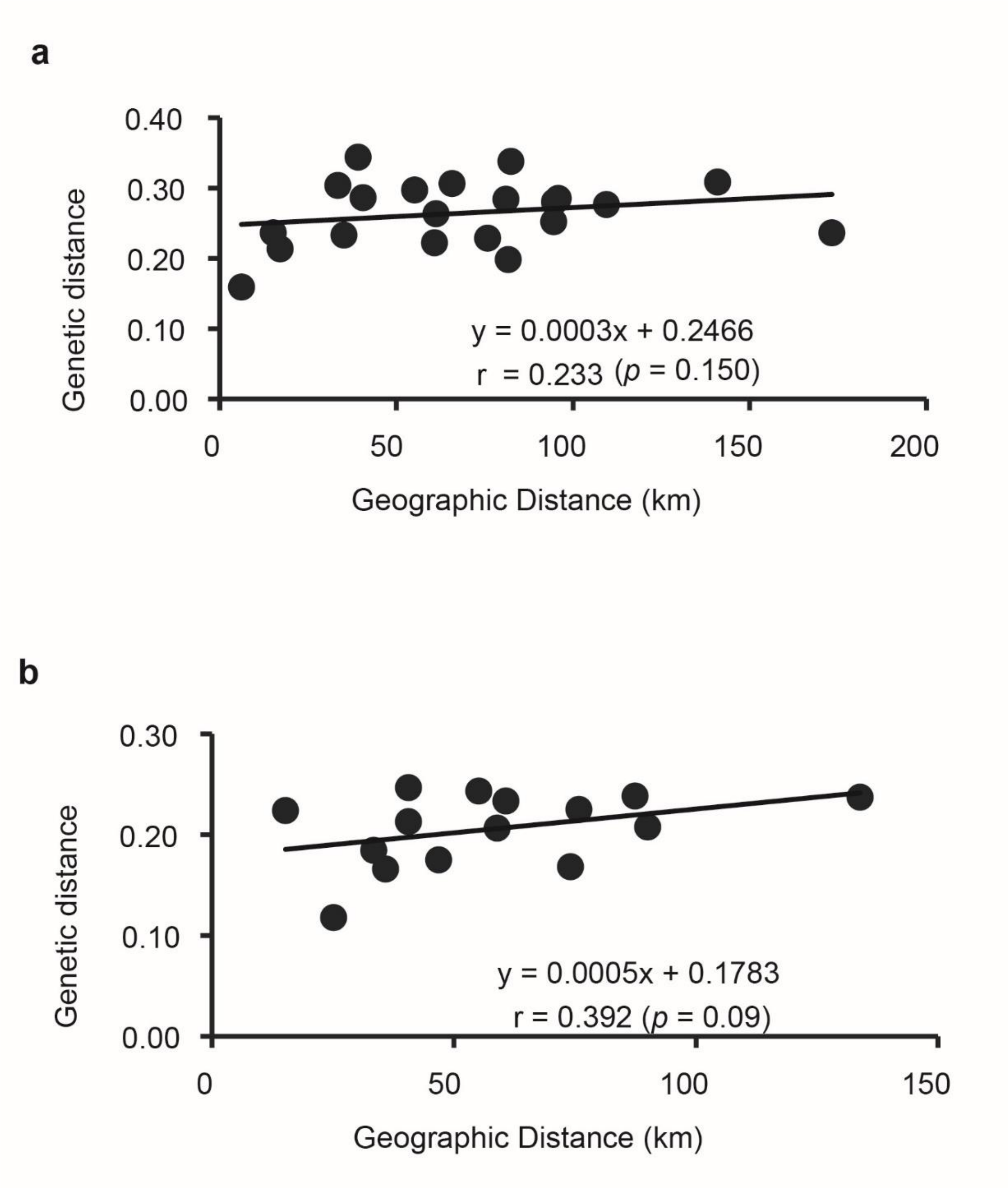

Supplement: Supplementary file 1 — Supplementary Information [file 41598_2019_54526_MOESM1_ESM.pdf]
